# Supplementary material for: Content-rich biological network constructed by mining PubMed abstracts
Source: BMC Bioinformatics. 2004 Oct 8;5:147. doi: 10.1186/1471-2105-5-147 (PMC528731; doi:10.1186/1471-2105-5-147)
Supplement: Additional File 2 — The original results of the above study (non-essential files are deleted to keep the file size under the limit set by BMC bioinformatics). [file 1471-2105-5-147-S2.bz2 › chilibotAdditionalFile2/dip05/643ID11106733E11030/html/HSLU_HSLV.html]

 


 **HSLU** and **HSLV** 
  
Found 35 abstracts in PubMed, retrieved 05.  
 

 What does Google say? 
 PDF only 
| .edu only 

---

**Interactive relationship** (e.g. stimulation, inhibition, etc)

**Neutral relationship**- In the prokaryotic homolog of the eukaryotic proteasome, HslUV, the double donut  **HslV**  protease is allosterically activated by  **HslU** , an AAA protein of the Clp Hsp100 family consisting of three amino terminal, carboxy terminal, and intermediate domains.  Ref: 12823960 J Mol Biol, 2003
- HslVU is a bacterial homolog of the proteasome, where  **HslV**  is the protease that is activated by  **HslU** , an ATPase and chaperone.  Ref: 12032294 Proc Natl Acad Sci U S A, 2002
- and the structure has been solved to 2.5A resolution, revealing an assembly in which a  **HslU**  DeltaI hexamer binds one end of the  **HslV**  dodecamer.  Ref: 12823960 J Mol Biol, 2003
- The conformation of the protomers of the  **HslU**  DeltaI complexed  **HslV**  hexamer is similar to that in the symmetric wild type HslUV complex, while the protomer conformation of the uncomplexed  **HslV**  hexamer is similar to that of  **HslV**  alone.  Ref: 12823960 J Mol Biol, 2003
- On the basis of the structure of a HslUV complex, a mechanism of allosteric activation of the  **HslV**  protease, wherein binding of the  **HslU**  chaperone propagates a conformational change to the active site cleft of the protease, has been proposed.  Ref: 12054822 J Mol Biol, 2002
- Here we report site directed mutagenesis, peptide activation, and fluorescence experiments that strongly support the functional relevance of the C terminus insertion mechanism we find that mutations in  **HslV**  that disrupt the interaction with the C termini of  **HslU**  invariably lead to inactive enzyme.  Ref: 12032294 Proc Natl Acad Sci U S A, 2002
- Conversely, synthetic peptides derived from the C terminus of  **HslU**  bind to  **HslV**  with 10 5 M affinity and can functionally replace full  **HslU**  particles for both peptide and casein degradation but fail to support degradation of a folded substrate.  Ref: 12032294 Proc Natl Acad Sci U S A, 2002
- Functional interactions of  **HslV**  ClpQ with the ATPase  **HslU**  ClpY.  Ref: 12032294 Proc Natl Acad Sci U S A, 2002
- The Escherichia coli and T. maritima  **HslU**  proteins mutually stimulate  **HslV**  proteins from both sources, suggesting a conserved activation mechanism.  Ref: 12646382 Biophys Chem, 2003

**Non-interactive relationship** (e.g. studied together, co-existance, homology, etc.)

- Crystal structure of HslUV complexed with a vinyl sulfone inhibitor corroboration of a proposed mechanism of allosteric activation of  **HslV**  by  **HslU** .  Ref: 12054822 J Mol Biol, 2002
- A feature in the Haemophilus influenzae HslVU structure, insertion of the C termini of  **HslU**  into pockets in  **HslV** , was not seen in all other structures of the enzyme.  Ref: 12032294 Proc Natl Acad Sci U S A, 2002
- an asymmetric  **HslU**  DeltaI 6  **HslV**  12 complex has been crystallized.  Ref: 12823960 J Mol Biol, 2003
- High similarities > or =57.5% with the prototypical  **HslV**  and  **HslU**  from Escherichia coli and conservation of residues essential for biochemical activity suggested that a functional HslVU complex is present in trypanosomatid protozoa.  Ref: 12446803 Mol Biol Evol, 2002
- The structure of the N termini of  **HslV**  and  **HslU**  further suggested mitochondrial localization.  Ref: 12446803 Mol Biol Evol, 2002
- Reaction in the crystals with a vinyl sulfone inhibitor reveals that the  **HslU**  DeltaI complexed  **HslV**  hexamer is active, while the uncomplexed  **HslV**  hexamer is inactive.  Ref: 12823960 J Mol Biol, 2003
- It consists of  **HslV** , the protease, and  **HslU** , the ATPase and chaperone.  Ref: 12646382 Biophys Chem, 2003
- These results confirm that  **HslV**  can be activated by binding of a hexameric  **HslU**  DeltaI 6 ring lacking the I domains, that activation is effected through a conformational change in  **HslV**  rather than through alteration of the size of the entry channel into the protease catalytic cavity,.  Ref: 12823960 J Mol Biol, 2003
- We showed that genes homologous to eubacterial  **HslV**  ClpQ and  **HslU**  ClpY are present in the genome of trypanosomatid protozoa and are expressed.  Ref: 12446803 Mol Biol Evol, 2002
- Comparison of the structure of the HslUV inhibitor complex with that of  **HslV**  without inhibitor and in the absence of  **HslU**  reveals that backbone interactions would correctly position a substrate for cleavage in the HslUV complex, but not in the  **HslV**  protease alone, corroborating the proposed mechanism of allosteric activation.  Ref: 12054822 J Mol Biol, 2002
- Structure and Reactivity of an Asymmetric Complex between  **HslV**  and I domain Deleted  **HslU** , a Prokaryotic Homolog of the Eukaryotic Proteasome.  Ref: 12823960 J Mol Biol, 2003
- Eubacterial  **HslV**  and  **HslU**  subunits homologs in primordial eukaryotes.  Ref: 12446803 Mol Biol Evol, 2002
- Phylogenetic analysis indicated that  **HslV**  and  **HslU**  from trypanosomatids clustered with eubacterial homologs but did not point to any particular bacterial lineage.  Ref: 12446803 Mol Biol Evol, 2002
